# Seven‐year‐olds’ aggressive choices in a computer game can be predicted in infancy

**DOI:** 10.1111/desc.12576

**Published:** 2017-07-24

**Authors:** Dale F. Hay, Mark K. Johansen, Peter Daly, Salim Hashmi, Charlotte Robinson, Stephan Collishaw, Stephanie van Goozen

**Affiliations:** ^1^ School of Psychology Cardiff University UK

## Abstract

Concerns about the relationship between computer games and children's aggression have been expressed for decades, but it is not yet clear whether the content of such games evokes aggression or a prior history of aggression promotes children's interest in aggressive games. Two hundred and sixty‐six 7‐year‐old children from a nationally representative longitudinal sample in the UK played a novel computer game (CAMGAME) in which the child's avatar encountered a series of social challenges that might evoke aggressive, prosocial or neutral behaviour. Aggressive choices during the game were predicted by well‐known risk factors for aggressive conduct problems and the children's own early angry aggressiveness as infants. These findings suggest that children who are predisposed to aggression bring those tendencies to virtual as well as real environments.

## RESEARCH HIGHLIGHTS

- Our study tested the hypothesis that children's aggression in the context of computer games can be predicted by their own aggressive tendencies years earlier.
- We created a novel computer game with embedded social challenges that could elicit aggression or alternative behaviour.
- Children's angry aggressiveness in infancy predicted their aggressive choices in the game at age 7 years.
- Angry aggressiveness in infancy also predicted how often children played games but that did not explain the link between early aggressiveness and aggressive choices.

## 1 INTRODUCTION

Concerns about the relationship between computer games and children's aggression have been expressed for decades (e.g., Silvern & Williamson, 1987). It is increasingly acknowledged that spending more time playing video games does not necessarily lead to violent behaviour (Cunningham, Engelstatter, & Ward, 2016; DeCamp, 2015), and so the focus for research has now turned to possible links between game‐playing and milder forms of aggression (Ferguson, 2015). However, even this claim has been met with scepticism (e.g., McCarthy, Coley, Wagner, Zengel, & Basham, 2016).

Two opposing causal models – exposure effects versus selection effects – have been offered to explain a correlation between game‐playing and aggression. Advocates of exposure effects propose that high levels of game‐playing might foster aggressive tendencies via the interplay of arousal within the game context and social learning processes, for example, identification with violent game avatars which might lead to higher rates of aggression in and out of game contexts (e.g., Anderson & Bushman, 2002). For example, young people in a large national cohort sample who played video games with violent content were at a somewhat increased risk for conduct disorder in adolescence (Etchells, Gage, Rutherford, & Munafó, 2016); however, playing violent video games was not associated with violent behaviour in a study that controlled for the adolescents’ social environment (DeCamp, 2015). Longitudinal analysis of the UK Millennium Cohort Study revealed that conduct problems were predicted not by the time children spent playing computer games but rather by the hours spent watching television (Parkes, Sweeting, Wight, & Henderson, 2013).

Alternatively, advocates of selection effects propose that a pre‐existing disposition to be aggressive, possibly underpinned by neurobiological processes, might heighten an individual's interest in computer games, particularly violent ones (e.g., Elson & Ferguson, 2014). For example, in a representative sample of German adolescent gamers, physical aggression predicted the use of violent video games one year later, but the reverse was not true, even when controlling for sex, education and frequency of video game play (Breuer, Vogelgesang, Quandt, & Festl, 2015). Tests of these alternative models require not just evidence for the influence of exposure to computer games but also suitable controls for potentially confounding factors.

In contexts where the child might make different choices in a game, children with a history of anger and aggression may be more likely to enact violent solutions to the dilemmas posed by the game. It is therefore important to obtain pretest information about children's prior aggressiveness before concluding that game‐playing makes children more aggressive. Longitudinal studies are required. However, to be convincing, longitudinal analyses must begin very early in development; the pathway to problematic levels of aggression originates in the first 2 years of life (e.g., Alink et al., 2006; Côté, Vaillancourt, LeBlanc, Nagin, & Tremblay, 2006; Lorber, Del Vecchio, & Smith Slep, 2015; NICHD Early Child Care Research Network, 2004), long before children develop the motor skills to play computer games.

Our longitudinal study of a representative British sample has demonstrated that individual differences in anger and the use of physical force emerge in the first year of life and predict aggression in childhood (Hay, Waters, et al., 2014). We hypothesized that individual differences originating in infancy might also predict children's later aggressiveness in virtual environments, even when controlling for other risk factors for early aggression and subsequent exposure to computer games. Therefore, as part of a battery of measures for an assessment of our longitudinal sample at 7 years of age, we created a bespoke first‐person perspective game in which the protagonist avatar encounters a number of social challenges from game characters that might evoke aggression or alternative responses. The fact that all the children in the sample were assessed in infancy and tested on a standardized game in middle childhood made it possible to control for early aggressive tendencies, prior to any experience of playing games.

If children's aggressive choices in the game could be predicted by their angry aggressiveness in infancy, long before they could physically play computer games, these analyses would provide strong evidence for selection effects. We controlled for key risk factors that were earlier found to be associated with aggression in infancy, in particular socioeconomic disadvantage in the family, parental aggression and mothers’ depression in pregnancy (Dodge, Coie, & Lyman, 2006; Tremblay et al., 2004; Waters, Hay, Simmonds, & van Goozen, 2014), as well as the children's current exposure to computer games.

## 2 METHOD

### 2.1 Participants

Recruitment into the Cardiff Child Development Study (CCDS) was approved by the Cardiff University School of Psychology Research Ethics Committee and the NHS Multi‐Centre Research Ethics Committee. Based on initial power calculations, 332 primiparous women were recruited from antenatal services in two National Health Service (NHS) Health Care Trusts in Wales, UK (see Hay et al., 2014). The recruitment strategy yielded a nationally representative sample, not significantly different on socio demographic characteristics from the families of firstborn children in the most recent UK national cohort study (Connolly & Platt, 2014; Kiernan, personal communication, 2009). By 7 years of age, 22 families had withdrawn from the study and one had never been traced, leaving 309 (93%) available for study. Of those, 287 (93%) were assessed at age 7. The current analysis focuses on 266 children (93% of those assessed at 7 years) who played a bespoke computer game designed to allow aggressive or alternative choices in response to social dilemmas. Sociodemographic characteristics are reported in Table 1.

**Table 1:** Demographic characteristics of the sample during the pregnancy

Variable	Total sample *N* = 332	Subsample *N* = 266
Mother's Age at Birth (Mean)	28.1	28.5
Stable Partnerships	90.4%	93.3%
Marital Status (% married)	52.9%	52.3%
Ethnicity (% British or Irish)	92.7%	93.3%
Social Class (% middle class)	50.9%	54.5%
Mother's Education (% > basic qualifications)	80.0%	80.8%
Child's Sex (% female)	43.0%	43.2%
Social Adversity Risk Factor Mean	.00	−.07

Note

Basic educational qualifications are defined as 5 or more pass marks on the national GCSE examinations in the UK or their equivalents. The socioeconomic adversity score for the subsample did not differ significantly from the overall sample recruited in pregnancy.

### 2.2 Materials

The *Castell Arth Mawr Adventure Game* (CAMGAME) is a first‐person perspective game inspired by the classic Robbers Cave experiment (Sherif, Harvey, White, Hood, & Sherif, 1949) which induced conflict between two groups of children attending a summer camp. The game script, which was approved by the Cardiff University School of Psychology Research Ethics Committee, was instantiated in a modified version of the game *The Elder Scrolls V: Skyrim* (Bethesda, 2011) using freely available development tools for that game (see Supplementary Materials). Children were asked to imagine they were on a school trip to a Welsh castle, accompanied by a teacher and classmates who were wearing red sweatshirts with their school logo. As they explored the castle, they discovered that strange children from another school, wearing blue sweatshirts, were also exploring the castle. The red‐shirted and blue‐shirted school groups were competing to find treasure hidden by the Bear King somewhere in the castle. Several challenges that might provoke aggression (taunts and shoving by the unfamiliar peers) and empathy (distress or injury shown by another person) were embedded into the script.

In this first‐person game, each player saw his/her own pair of hands picking up a mallet that could pound things or people. The mallet could be used prosocially to respond to requests for help made by characters in the game or aggressively to hit other people.

### 2.3 Procedure

Parents were interviewed at home and completed demographic questionnaires during the third trimester of pregnancy. The children were assessed at mean ages of 6, 12, 21 and 33 months in an alternating sequence of home and laboratory visits, with two home visits at age 7. One postgraduate researcher interviewed the parent and another tested the child on measures of cognition, language and social problem‐solving, as well as the CAMGAME.

### 2.4 Measures

#### 2.4.1 Socioeconomic adversity

A general index of child's exposure to maternal factors known to be associated with risk for social adversity was created using polychoric principal component analysis. The maternal experiences that contributed to this index were: (1) the mother not having achieved basic educational attainments (i.e., the mother having no qualifications or fewer than five GCSEs or equivalent attainments); (2) the mother being 19 years of age or under at the time of child's birth; (3) the mother not being legally married during the pregnancy; (4) the mother not being in a stable couple relationship during the pregnancy; and (5) the mother's current occupation being classified as lower status according to the Standard Occupational Classification 2000 (SOC2000; Elias, McKnight, & Kinshott, 1999). All the items contributed to a single component (eigenvalue 3.84) which explained approximately 77% of the shared variance. Summary factor scores derived from the analysis were used as a proxy for socioeconomic adversity.

#### 2.4.2 Mothers’ antisocial behaviour

Mothers reported on their history of arrest as part of the life events section of the Wave 1 questionnaire. The questionnaire battery at Wave 1 also included a section labelled ‘What I Am Like’, which included items from the screening questionnaire for the International Personality Disorder Examination (IPDE; Loranger et al., 1994). The screening questionnaire associated with the IPDE interview has been used in the UK and in community samples, including a large national sample in Australia (Lewin, Slade, Andrews, Carr, & Hornebrook, 2005). For the present analyses, a subset of IPDE screening items that corresponded to the DSM‐IV criteria for Antisocial Personality Disorder (ASPD) was identified along with an additional set of items measuring DSM‐IV symptoms of CD were included in the questionnaire. The conduct symptom items were incorporated into a section of the Wave 1 questionnaire entitled ‘What I Was Like as a Child’. A composite variable created by summing the two individual scales showed an acceptable level of internal consistency, α = .79, and was further validated by mothers’ reports of their history of arrest, point biserial *r* (323) = .56, *p* < .001.

#### 2.4.3 Prenatal depression

The Wave 1 prenatal interview incorporated the mood disorder and anxiety disorder sections of the Schedules for Clinical Assessment in Neuropsychiatry (SCAN; Wing et al., 1990), with an additional screen for psychotic symptomatology. Final decisions about clinical diagnoses were made in case conferences with two adult psychiatrists. There was significant agreement between two psychiatrists’ diagnoses of prenatal disorder, κ = .78, *p* < .001, and past disorder, κ = .76, *p* < .001. Dichotomous variables were created to measure presence of DSM‐IV mood disorder (Major Depressive Disorder or Bipolar Disorder) in pregnancy. Individual cases were reviewed to exclude individuals presenting in pregnancy with manic symptoms only.

#### 2.4.4 Angry aggressiveness in infancy

At 6 months postpartum, up to three informants per family (mothers, fathers, and a third family member or friend) completed a developmental milestones checklist that included a brief four‐item scale, the Cardiff Infant Contentiousness Scale (CICS), which was designed to assess infants’ explicit expressions of anger and use of force against their companions (Hay et al., 2010). The four items that comprise the CICS scale are *hits out at people*;* angry moods*;* bites*; and *has temper tantrums*. The CICS scale had adequate internal consistency (median α across mothers, fathers, and third informants being .69), with significant agreement between all possible pairs of informants, with high agreement between parents, *r* (217) = .51, *p* < .001. The CICS ratings were validated by direct measures of the infant's distress when confined, that is, strapped into a car seat and observed tendencies to strike out at or grab toys from peers (Hay et al., 2010) and predicted the children's later aggressive conduct problems at age 3 years (Hay et al., 2014).

CICS factor scores were derived through a measurement model using Mplus 7 whereby a latent dimensional construct was estimated using the maternal, paternal and third informant's assessments as indicators. These factor scores, analogous to standardized scores, were constrained to have a mean of 0 and *SD* = 1. Mplus 7 (Muthén & Muthén, 2012) was used to implement this measurement model and calculate a factor score.

#### 2.4.5 Time spent playing computer games

At age 7, as part of a clinical interview, the Preschool Age Psychiatric Assessment (PAPA; Egger et al., 2006), the parent was asked about the child's usual activities, including the estimated time spent playing computer games. The interview yielded an ordinal variable ranging from never to three hours or more per day. The variable was dichotomized to contrast those children who played computer games daily versus other members of the sample.

#### 2.4.6 Children's aggressive choices in the CAMGAME

Five events that might evoke aggressive choices were embedded into the game (Table 2). In each case, the child's use of the mallet against game characters was used as an operational definition of an aggressive choice, which was given a score of 1. Testers recorded the child's response to each event on an IPad. Scores from each challenge were summed (range, 0 to 5), yielding a scale of aggressive response to challenges in the game that showed internal consistency (α = .69) across five vignettes in the game. The proportion of aggressive choices was computed for each child and an arc sine transformation of those proportion scores was used.

**Table 2:** Vignettes in the game scored for aggressive responses

Event	Vignette	Aggressive choices	Prosocial choices
First Push Scene	In the distance, in front of the door leading to the next scene, there is a child from another school. As X walks up the path, X's friends from the red school say, ‘*Who's that?*’ and ‘*He's from that blue school!*’ When X approaches, the blue school child says, ‘*You red school loser!*’ and X is pushed away.	X uses the mallet to hit the blue school child	N/A
Storyteller Scene	X enters the scene and walks towards an elderly character, the Storyteller, who says, ‘*Hello children I am the Storyteller! This was the castle of the Bear King, before he left the buried treasure in the caves! If you're quick you might find it, before those blue school kids do*.’	X uses the mallet to hit the Storyteller	
	As X proceeds through this scene to the door, the Storyteller says she is cold and asks, ‘*Before you go, could you help me? Could you hit that woodpile with your mallet? I need it for later to warm up*.’		X uses the mallet to hit the woodpile to help the Storyteller build a fire
Cave Scene	X enters the scene and is pushed back by the blue school children, who say, ‘*You red school loser!*’ and ‘*I pushed you that means go away!*’ X's friends from the red school are on the other side of the room. One of the children is on the floor and says, ‘*Ow! They pushed me!*’ If X approaches the blue school children, X is further taunted, ‘*Yeah we pushed you and your friends, so what? We're gonna get the treasure before you*.’ If X approaches the red school friend, X hears, ‘*Oh thank you, I'll be okay*.’	X uses the mallet to hit the blue school child	X walks over to the injured red school friend
Ditch Scene	X walks along the path and hears the red school friends say, ‘*Those mean bullies pushed us down here*.’ The blue school children are at the end of the path whilst the red school friends are at the bottom of a staircase. As X moves closer to the blue school children they say, ‘*Oh look, it's stupid again*.’	X uses the mallet to hit the blue school child	X walks to injured red school friend
Racing to the Treasure Scene	In this final scene, X is trying to find the entrance to the castle. X encounters the red school friends and the blue school children who are also ‘racing’ to the entrance. There is no taunting speech from the blue school children in this scene.	X uses the mallet to hit the blue school child	N/A

A further check on the validity of the child's use of the mallet as a measure of aggression against game characters was made by examining mallet use in two vignettes that were directed to inanimate objects (smashing bottles in the absence of any requests for help), not child or adult characters. A principal components analysis showed that use of the mallet for bottle‐smashing loaded on a separate factor from the five challenges that entailed aggression against human characters.

#### 2.4.7 Teachers’ ratings of children's aggressive behaviour problems

Children's current levels of aggression at age 7 were assessed by classroom teachers, using the 19‐item aggressive syndrome scale of Teacher Report Form (TRF: Achenbach & Edelbrock, 1986). Coefficient alpha for the TRF aggression scale was .95 in this sample.

## 3 RESULTS

Descriptive statistics and univariate correlations between the study variables are presented in Table 3. Correlations for girls are above the diagonal; correlations for boys are below the diagonal.

**Table 3:** Intercorrelations amongst study variables

	1	2	3	4	5	6	7
1. Socioeconomic adversity factor		.51***	.46***	.02	.31***	.26**	.06
2. Maternal antisocial behaviour	.47***		.44***	.02	.23**	.30**	.11
3. Prenatal depression	.44***	.33***		.06	.24**	.22*	.21*
4. Daily use of computer games	.10	.23**	.12		.32***	−.14	.02
5. Infant CICS score	.34***	.31***	.33***	.16+		−.06	−.01
6. Teachers’ aggression ratings	.28**	,11	.16+	.11	.01		.13
7. Aggressive choices in game	.08	.10	.23**	.08	.22**	.11	
Mean	.00	4.43	.16	.49	.00	2.88	.20
*SD*	1.00	4.09	.37	.50	.86	5.83	.24

Note

^+^ *p* < .10; *p < .05; **p < .01; ***p < .001.

Correlations for girls and boys are presented above and below the diagonal, respectively.

### 3.1 Aggressive choices in the game context

The percentage of children who hit human characters ranged from 34% in response to the unfamiliar children's taunts or pushing to 13% in response to the request for help from an old woman. A slight majority of children (51%) never made an aggressive choice.

The mean proportion of aggressive choices across the challenges was .19 (*SD* .23), ranging from 0 to .8. The proportion of aggressive choices in the game was significantly associated with teachers’ ratings of children's aggressive behaviour problems, *r* (244) = .19, *p* = .002. On average, girls’ aggression scores (*M* = 0.08, *SD* = .15) were significantly lower than those of boys (*M* = .27, *SD* = .24), *t*(257.4) = −8.17 *p* < .001. To meet the assumptions of subsequent analyses, the aggressive choice variable was dichotomized, contrasting those children who made an aggressive choice no more than once (*N* = 185) with those who showed aggression more than once (*N* = 81).

### 3.2 Early prediction of aggressive choices in the game

The first set of analyses tested for links between angry aggressiveness in infancy and aggressive choices in CAMGAME, while controlling for the key risk factors of socioeconomic adversity, maternal aggressiveness, prenatal depression, and male sex, which at the univariate level had been found to be correlated with the infants’ angry aggressiveness (Hay et al., 2014). These risk factors were entered at the first step of a logistic regression model, with the child's angry aggressiveness in infancy added at the second step. The analysis revealed that, even when the risk factors were taken into account, angry aggressiveness in infancy predicted the children's aggressive choices in the computer game at age 7 (Table 4).

**Table 4:** Very early prediction of aggressive choices in the *Castell Arth Mawr Adventure Game* at age 7 from early risk factors and angry aggressiveness in infancy

Predictor	B	*SE*	Wald	*df*	*p*	*EXP(B)*	95% CI	
							Lower	Upper
Adversity	−.32	.21	2.33	1	.13	.73	.48	1.09
Mother antisocial	.03	.04	.55	1	.46	1.03	.95	1.12
Prenatal depression	.97	.47	4.26	1	.04	2.64	1.05	6.62
Male gender	2.03	.37	30.41	1	.0001	7.62	3.70	15.69
Early CICS score	.45	.19	5.42	1	.02	1.56	1.07	2.28

Note

Coefficients in the table are those obtained at the final step of the logistic regression model. The dependent variable is 2+ vs. 0/1 aggressive choices in the game. Nagelkerke *R* ^2^ = .26.

### 3.3 Did frequency of game‐playing account for children's aggressive choices in the game?

At age 7, the vast majority of children (88.1%) were reported to enjoy playing computer games; 41% of girls and 55% of boys played games daily and were therefore classed as frequent users of computer games. Angry aggressiveness in infancy was significantly associated with the children's daily use of computer games at age 7, *r* (255) = .24, *p* < .001. This suggests that the link between angry aggressiveness and aggressive choices might be explained by current exposure to computer games. However, in a follow‐up analysis, after controlling for the daily use of computer games, early aggressiveness remained a significant predictor of the children's aggression in the context of CAMGAME, Wald statistic = 8.69, *p* = .003, Exp(B) = 1.63 (95% CI = 1.18 to 2.26) and daily use of games was not significantly associated with the children's aggressive choices, Wald statistic = .92, *p* = .34.

## 4 DISCUSSION

The aim of our study was to examine children's aggressive choices in a computer game, as a way to tap into the moment‐to‐moment decisions they make in response to social dilemmas that could provoke aggression. Our findings suggest that those children in a representative community sample who showed early signs of anger and aggression were subsequently likely to spend more time playing computer games and to make aggressive choices in those games. Our findings are relevant to the hypothesis that aggressive children opt to play aggressive games, rather than the games themselves making children more aggressive (Elson & Ferguson, 2014). Our longitudinal design and assessment of infants’ signs of anger and use of force prior to developing the motor skills needed for the playing of computer games provided a unique opportunity to conduct a strong test for selection versus exposure effects.

As expected in middle childhood, boys made more aggressive choices in the game than girls did. However, when gender was taken into account, prenatal exposure to the mothers’ depression was the key family risk factor for aggressive choices in the game, just as it is a known risk factor for anger, aggression and violent crime (Waters et al., 2014).

In this study, the children played a bespoke game that was not designed to be violent and therefore extremely aggressive behaviour was not modelled by characters in the game. Rather, the children were responding to fairly mild provocations – for example, being bumped into or taunted by peer avatars. In some instances, they responded with aggression rather than prosocial behaviour to game characters’ requests for assistance. It would be of interest to see whether prior risk factors and early aggressiveness also predicted aggressive choices in a more violent game where aggressive actions were more explicitly modelled.

Our study has limitations. Assessment of the children's exposure to games relies on one parent's report. Only one game was played. As in much of the literature on computer games (Ferguson, 2015), effect sizes are relatively small. Situational factors, motoric competence, and expertise in the playing of computer games are likely to influence the children's playing of the game. However, the findings are in line with other evidence for selection effects in the literature on game‐playing (e.g., Breuer et al., 2015) and extend other research that draws attention to the early origins of an aggressive approach to the social world (e.g., Alink et al., 2006; Baillargeon et al., 2007; Breuer et al., 2015; Côté et al., 2006; NICHD Early Child Care Network, 2004). Early aggressive tendencies predict behaviour in virtual as well as real environments.

Our findings suggest that the emotional and decision‐making processes underlying the deployment of aggression might be probed using such virtual environments, such as the novel game paradigm created for this research. Furthermore, aggressive children's attraction to computer games opens up possibilities for future game‐based intervention strategies to reduce aggressive decision‐making.
